# Meager Perception of Preconception Care Among Women Desiring Pregnancy in Rural Areas: A Qualitative Study Using Focus Group Discussions

**DOI:** 10.3389/fpubh.2021.689820

**Published:** 2021-10-15

**Authors:** Prakash Prabhakarrao Doke, Jayashree Sachin Gothankar, Prasad Dnyandeo Pore, Sonali Hemant Palkar, Amruta Paresh Chutke, Archana Vasantrao Patil, Aniruddha Vinayakrao Deshpande, Khanindra Kumar Bhuyan, Madhusudan Vaman Karnataki, Aparna Nishikant Shrotri

**Affiliations:** ^1^Bharati Vidyapeeth Deemed University, Pune, India; ^2^Government of Maharashtra, Mumbai, India; ^3^UNICEF United Nations International Children's Emergency Fund, New York, NY, United States

**Keywords:** knowledge, behavior, preconception care, food habits, height and weight, tobacco consumption, alcohol consumption, Maharashtra

## Abstract

**Background:** India has the second-highest number of under-five deaths in any country in the world. WHO and the Government of India recommended the rollout of preconception care (PCC) to reduce maternal and child mortality. However, very few countries, including India, have started a comprehensive package of PCC services. It implies that women, mainly from rural and tribal areas, are not aware of PCC. PCC has been rolled out through the government health system in two blocks of Nashik district in Maharashtra state, India, among all women who desire to be pregnant within 1 year. This project is the first of its kind in India. To assess basic perceptions, knowledge, and behavior of women on PCC before the implementation of the project, focus group discussions (FGDs) were carried out. The authors think that the finding may help to develop strategies for behavioral change communication.

**Methods:** From each of the four blocks, two villages having subcenter were selected for conducting FGD. A house-to-house survey was conducted by Accredited Social Health Activist (ASHA) to enlist women who desire a baby in 1 year and invite them to subcenter for FGDs, which were conducted in June 2018.

**Results:** A total of 76 women having a mean age of 23.97 years participated in the FGDs. Most of them (46.05%) had completed 10 years of education. About 50% of pregnancies were planned. The decision about the timing of the first pregnancy is influenced by the mother-in-law. Women knew that they should not conceive before 20 years of age, and their suboptimal weight may have an adverse impact on the health of the newborn. There are many myths about food like “hot and cold foods” and “forbidden food” etc. Women had some knowledge about the adverse effects of tobacco and alcohol; very few consumed these. Most of them did not practice behaviors or accessed services related to PCC.

**Conclusions:** Women neither have the knowledge nor adopt behaviors or accessed services related to PCC. Roll out of PCC among them may help in further reduction of maternal and neonatal morbidity and mortality in India.

## Introduction

In India, the total number of deaths below 5 years of age reduced from 3.2 million in 1990 to 0.82 million in 2019. In 2019, India had the second-highest number of under-five deaths (1). There are wide inter and intrastate variations in under-five mortality and neonatal mortality (2). The common causes of neonatal mortality are preterm birth, birth asphyxia, and infection (3). The indirect causes are unplanned pregnancies, maternal undernutrition, pregnancies in adolescents, poor quality services, or inability to access care during the antenatal period, most notably intrapartum care and the neonatal period, etc. (4). Hence, WHO recommended the rollout of preconception care (PCC) in all the countries in 2013 (4). *Rashtriya Bal Swasthya Karyakram* Government of India published a report in 2018, which focused on creating awareness about PCC among couples. The commitment of the Government of India to the global community for ending preventable newborn death in India is articulated in the India Newborn Action Plan 2014, (5) based on Pan American Health Organization (PAHO) and WHO guidelines (6, 7). One of the six pillars of the India Newborn Action Plan is promoting PCC. However, PCC has not been rolled out systematically in India and most countries. Presently, PCC activities are limited to family planning and peri-conceptual folic acid administration. The coverage of peri-conceptual folic acid is low in most countries, and it is not captured in the routine health management information system. Behavioral changes constitute a major share of components of recommendations by WHO (4). The Government of Maharashtra, with the support of UNICEF, has started PCC in one tribal block (Peint) and one nontribal block (Sinnar) in Nashik district since 2018. Fourteen recommended interventions by PAHO are relevant for India (6). PCC package must address 13 categories of risk factors (4). The authors identified the following seven interventions for PCC implementation at the primary health-care level in these two blocks: enlisting of women who are planning pregnancies, clinical examination supported by laboratory testing, treatment, referral to higher facilities, prophylactic supplementation, monthly follow-up, and the overarching strategy of behavioral change communication using different media. The main criteria for the identification of these interventions were the magnitude of the problem in the community, cost, and feasibility of implementation at the primary health-care level.

The perception of health-related issues is determined by several factors. They may be grouped under biological (age and sex), socioeconomic (education, income, occupation, cultural practices, and past experiences), topographical, psychological factors, etc. Knowledge is one of the strong determinants. The assessment as well generation of perception is an intricate process. In the absence of any uniform PCC services in the public sector, the near absence of specialists in rural/tribal areas, low female literacy level, we felt that basic perceptions based on past experiences and culture could be recognized. These will be useful in deciding the contents of Behavior Change Communication (BCC) material and the resources required for the implementation of the PCC project.

To our knowledge, in India, this is the first qualitative study at the community level on preconception knowledge and behavior among women desiring pregnancy in near future.

The objectives of these focus group discussions (FGDs) were to find out whether women have any knowledge about PCC, to identify PCC-related behavior, and access the PCC services.

## Materials and Methods

Consolidated criteria for reporting qualitative research (COREQ) have been adopted to report the findings.

### The Research Team and Reflexivity

#### Personal Characteristics

Four faculties from the Department of Community Medicine and authors of the study conducted FGDs acting as moderators. They are postgraduates in community medicine, and one is Doctorate. Two moderators are male and two are females. The moderators have experience ranging from 15 to 42 years. Two research coordinators acted as facilitators and helped moderators. Both are women with postgraduation in public health.

#### Relationship With Participants

The moderators and facilitators met the participants for the first time during FGDs. However, health-care workers like Accredited Social Health Activist (ASHA) and Auxiliary Nurse Midwife (ANM) had briefed them about the authors and the study and the planned actions after the FGDs.

### Study Design

#### Theoretical Framework

The study comprised eight FGDs. After conducting FGDs, the authors carried out the content analysis.

#### Participant Selection

Trained ASHAs conducted a house-to-house survey using structured, validated, and pretested formats. ASHAs specifically asked each woman in the reproductive age group about her desire to have a child succeeding 1 year. A village-wise list of all such women was prepared. The authors randomly selected eight subcenter villages for conducting FGDs. ASHAs invited all eligible women for FGD and communicated to them the time and place of discussion. About 20% did not attend the discussions.

### Setting

**Figure 1:**
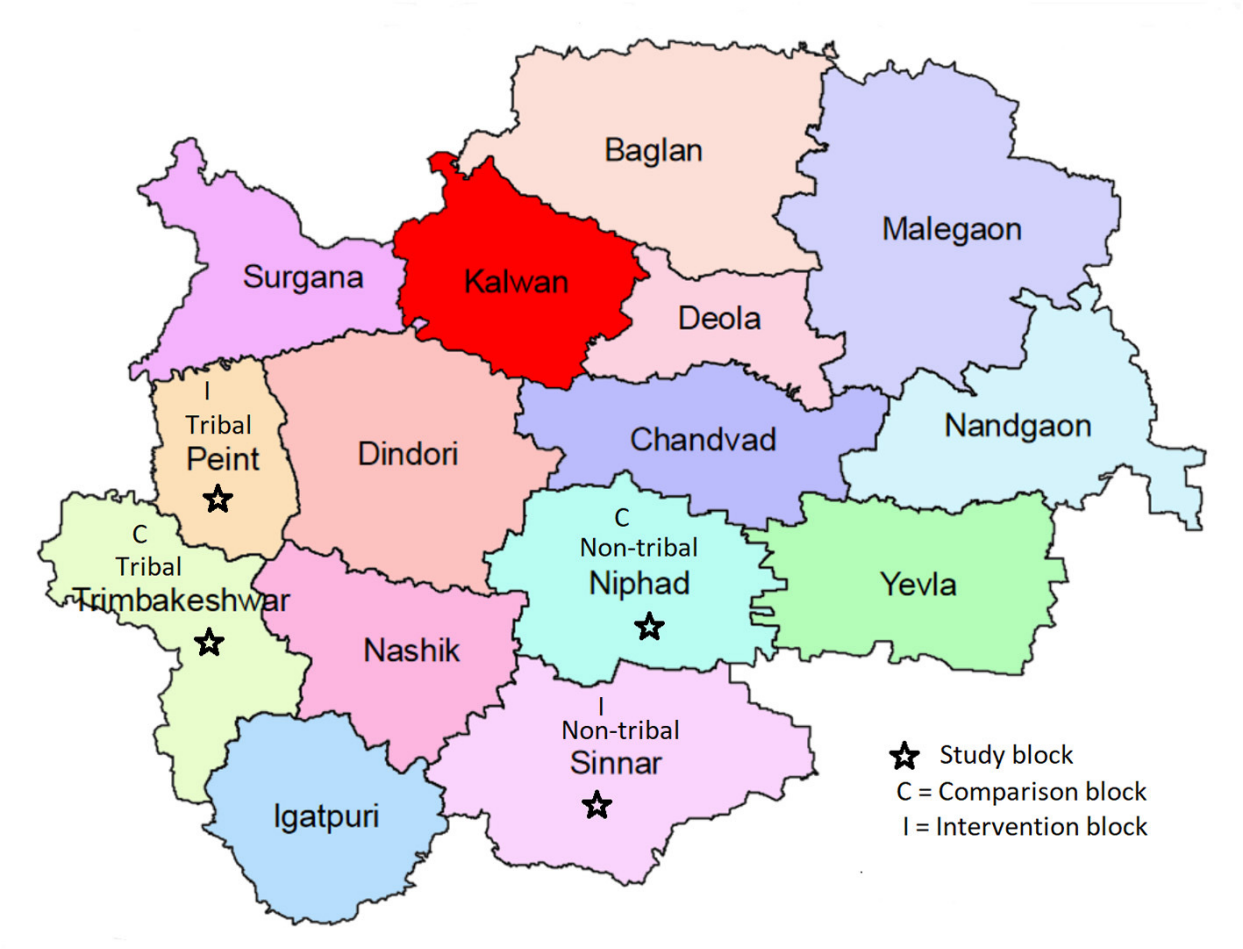
Blocks in Nashik district, India.

The study was conducted in Nashik district situated in north Maharashtra, India. The district has a population of 6,107,187 (census, 2011) scattered in 15 blocks. The proportion of the rural population in the district is 57.47%, inclusive of 31.55% tribal population. Before the intervention, four FGDs were conducted in these selected blocks (Tribal, Peint, and nontribal Sinnar) and an additional four control blocks (Tribal block, Trimbakeshwar, and non-tribal block Niphad). The total population of these four blocks is 976,149, of which the tribal population is 38.56%. The Tribal blocks have hilly terrain, suboptimal transportation facilities, and are away from urban areas. The literacy, particularly among women, is low. Blocks identified for the study are depicted in Figure 1.

The information like literacy rate, number of villages in these blocks, number of villages selected for FGDs with the number of households, and population in these villages are given in Table 1.

**Table 1:** Blocks and villages in the study area, Nashik District, India, 2018.

**S. No**.	**Block**	**Female literacy rate %**	**Villages in the block**	**FGD number** **name of villages in which conducted**	**Households**	**Village** **population**
1	Peint (T)	53.34	145	1. Bhaygaon 2. Gonde	298 254	1,447 1,276
2	Sinnar (NT)	48.93	131	3. Kundewadi 4. Datli	284 429	1,959 2,356
3	Trimbakeshwar (T)	63.93	126	5. Torangan, 6. Shirasgaon	367 280	1,984 1,795
4	Niphad (NT)	66.34	137	7. Chitegaon 8. Kothure	881 1,022	4,616 5,022
	Total	61.19	539	8	3,815	20,455

*FGD, Focus group discussion; T, Tribal; NT, Nontribal*.

The FGDs were conducted in subcenter buildings. The ANMs of the subcenters were not involved. The age group and education of participants are given in Tables 2, 3. The FGDs were conducted in June 2018.

**Table 2:** Age of the women participating in FGD, Nashik, India, 2018.

**Age group (years)**	**Number**	**%**
15-19	5	6.58
20-24	42	55.26
25-29	21	27.63
30-34	7	9.21
35-39	1	1.32
Total	76	00.00

*Mean age = 23.97 years (SD = 4.12); range 18 to 39 years*.

**Table 3:** Educational status of the woman participating in FGD, Nashik, India, 2018.

**Educational status**	**Number**	**%**
Illiterate	3	3.95
Up to primary (1st−7th std.)	12	15.79
Up to secondary- SSC (10th)	35	46.05
Up to HSC (12th)	17	22.37
Up to graduation	9	11.84
Total	76	100.00

*SSC, Secondary School Certificate; HSC, Higher Secondary Certificate*.

### Data Collection

The authors, through the consultative process, prepared a guide for conducting FGDs. The authors first validated and pretested it in an area that was not a part of the study. It had the following themes.

1. Planning of pregnancy
2. Age of the woman
3. Height and weight of the woman
4. Physical work
5. Nutrition
6. Tobacco and alcohol consumption
7. Medical/health assistance before pregnancy
8. Preconception services

The moderators had interactions with the women once only. All the communication was in the local language (Marathi). In each FGD initially, the moderator explained the purpose of the meeting and appealed active participation in the discussion. Their written informed consent was obtained for participation, audio recordings, and subsequent publication. The numbered placards were given to each participant to avoid personal identities. The authors encouraged the non-speaking women to speak. The discussion was initiated by few words about subthemes. Further probing was done if the discussion was digressed. All procedures were recorded. The facilitator also took written notes. The average time taken for one FGD was about 55 min. The transcripts were read and heard many times before translation into English. The transcripts are deposited in the department and UNICEF.

### Ethical Considerations

Approval from the Institutional Ethics Committee was obtained before the initiation of the study. Authors obtained informed consent for participation and publication from all the women. All personal identifiers were removed before the data analysis; only de-identified data were analyzed.

## Results and Analysis

The number of participants in the FGDs ranged from 6 to 12. More than 92% of women were in the age group of 20 to 40 years. About 80% had school-level education. The number of participants and FGD number are given in bracket in the script while including specific quotes. Moderators observed that in most of the FGDs, and participants needed a little more persuasion to initiate the discussion. For all the FGDs, the discussions were initiated with an inquiry about the details of family members, including the number of children the participants had. This initial discussion helped to break the ice. The moderator initially led the discussion toward pregnancy care for a short time and then the perception of participants about the care before pregnancy. The analysis was done using MAXQDA. Two researchers independently coded themes and codes, and any discrepancy was discussed among researchers and resolved.

Following are the key observations that are documented during the FGDs as per the subthemes.

### Pregnancy Planning

It was a concern, and its decision was taken by the family rather than the couple. In-laws or elderly persons in the family expect a child from the couple immediately after the marriage. However, many times a couple wants to postpone the pregnancy, discontinue the unplanned pregnancy, but it is carried forward, and the right of a woman to decide about abortion or to make choices is not given any importance. “…no, the first baby was eight months old, and I had conceived that time, but my family did not support abortion. They said let anything happen, but not to do abortion as it is another life” (7/3).

However, it was observed that some couples are having a dialogue on pregnancy planning. Such a process of joint decision-making positively impacts the health and empowerment of women. Few women narrated their experiences. “I had asked my husband if we want a child or not. We decided that time, after check-up… now we don't want a second baby, let's see…” (2/8). It was also noted that some women are not having clarity on whether they should have a child or when to have it. This was observed among recently married women, including those educated. The reason may be the lack of empowerment for decision-making among married women despite being educated. In many cases, women are unable to express and execute their wish not to have a child. A woman wanted to join the school and was unwilling to have a child; however, she did not communicate this with her husband, and both of them were not using any contraceptives (2/2).

Another observation was that people do not have any gender preference when the woman has conceived for the first time; however, the second time, gender opposite of the first child is desired. Usually, in such cases, the first girl child is accepted; however, the pressure on the woman to have a son is more when she plans for a second child.

“I have the first daughter. Now, my family desires that there should be a son” (4/8).

“Son is preferred… my husband also wants a son” (1/8).

FGD facilitator: “Are you worried that you may not give birth to a son?”

“Yes, it is expected. It is good if there is a son…”(2/8). The pregnancy planning concept is not yet widespread or widely practiced in the community. Instead, the term “family planning” is understood as the spacing between two children or not having a child. This perception in the community could be a result of government efforts. Various contraceptive methods were introduced for population control under, “family planning program.” It was also observed that planning is not done for the first pregnancy; however, after the first child, spacing between the first and the second child is practiced.

Women narrated experiences of domestic violence if they are unable to give birth to a child. Contrarily, women think that violence stands as a barrier in conception.

### Concept of a Healthy Mother and a Healthy Baby

Women think that a healthy mother will deliver a healthy child. They feel that a child with a fair skin complexion looks chubby, has a minimum 3 kg weight at birth, does not have deformity, is healthy, etc. A woman said, “a healthy should understand the touch, his/her nutritional needs, and should have got mental development” (3/4).

Women also shared the concept of a full-term baby. “The baby should be born after completing 9 months and 9 days in the mother's womb… the baby needs to be kept in the glass box if the baby is born premature… the weight of the baby should not be less than 2.5 kg. preferably, it should be between 2.5 to 3.5 kg at birth…” (9/3). Participants also described the concept of a healthy mother in various ways. Few women knew that height is also a determinant of weight and some women have weight disproportionate to their height, concept of body mass index (BMI). According to them, if the weight of a mother is low, the child will be malnourished and will have low birth weight. Participants described the following criteria about a healthy mother.

- The age of a mother should be more than 18- to 20 years and less than 30 years; however, some women said an expecting mother should be of 24 years as they are matured to become a mother. “19 years is not the age to become a mother; at this age, the baby does not grow properly in her womb. She becomes weak after delivery” (3/7).
- The mother should have positive thinking during pregnancy.
- The mother should have sufficient blood in the body.

A woman ideally should conceive 2 years after her marriage because by then, she gets some information about caring for the child. Some women think that girls should not get married before 21; otherwise, they cannot complete their education. Women are generally unaware of the consequences of early marriage; they feel that it will cause some problems during delivery. “The gap between two children should be a minimum of 2 years. Because I think that a woman does not suffer more if the gap between the two is longer…” (2/4).

### Physical Work

Participants think that before conception rest is not needed, because inactivity may lead to obesity, which would result in the inability to conceive. Instead, the woman should rest well after she conceives. Apart from physical activity and diet, few women talked about the mental health of a pregnant woman. They also shared their experience that a woman having stress may have difficulty in conceiving.

### Nutrition/Diet Concepts

Concepts of “hot food” and “cold food” are prevalent as traditional knowledge of food and its close association with health is firmly rooted. Most of the women used the concept of a “balanced diet” for a pregnant woman. Only one woman mentioned Take-Home Ration (THR) food served from Anganwadi, which indicates the low awareness and utilization of government programs.

Traditionally, some women use iron vessels for cooking, which serves as a good source of iron, and its use has been documented for addressing iron deficiency, anemia. However, it was also observed that aluminum and steel vessels are commonly used for cooking. In contrast, an iron pan was popularly used to make roti (bread of wheat flour)/ bhakari (bread of sorghum millets), an Indian bread made of wheat or sorghum flour. The food items to be eaten or not be eaten by a pregnant woman or a woman who wants to conceive, mentioned by them, are given in Table 4. Apart from the list, women provided the following thumb rules to be followed by a pregnant woman:

- Must eat a minimum of three times a day.
- Must eat on time.
- Must not keep fast as it causes weakness.

**Table 4:** Perceptions of women about food items during preconception and pregnancy.

**Must eat food items**	**Don't eat food items**
Milk	Oily/ fried food items
Fruits	Spicy foods as they are considered to be hot food
Available green leafy vegetables, Shepu (Dill leaves), Chavali (black eyed beans)	Papaya and banana
Cereals	Salty food
Eggs	Eggs in summer as they are considered as hot food
Chicken	
Fruit vegetables	
Curd, buttermilk is cold and beneficial	
Dal Khichadi (a dish made of rice and lentils)	

### Tobacco and Alcohol Consumption

Most women said older women mainly use Mishri, and alcohol consumption is highly prevalent among men. Women are aware that cigarette causes cancer. They also said that if a pregnant woman consumes alcohol or cigarette, then it harms the fetus. A woman narrated an experience of a woman who used Mishri even during her pregnancy. Her fetus was found abnormal, and later it was aborted (5/8). Some of the women said that few women drink locally produced alcohol. Some women reported that “Older women consume tobacco.” Though they are unaware of the consequences of alcohol consumption, tobacco consumption on pregnant mothers and fetuses, they understand it is bad for the health of the child. Women think that it will cause an abnormality in the fetus or lead to abortion or die inside the womb.

### Health Services Before Pregnancy

Women seek health care before pregnancy only when they are unable to conceive despite trying. Women think that doctors can ascertain the need for conception treatment. For PCC, women enlisted the following problems/tests during a checkup:

- Calcium deficiency.
- Tablets to increase blood amount.
- Blood pressure.
- HIV test.
- Swine flu test.
- Hemoglobin.
- Blood sugar levels.
- Height and weight.
- Thyroid.
- Jaundice.
- Vaccination.
- Ultrasound.

Family decision plays a vital role for accessing health services. A woman cannot get treatment without the concurrence of her family. Most of the women think that care should be taken after conception. However, it was seen that very few women are aware of PCC, right from the planning of pregnancy, so that the child will be healthy. However, the majority were not aware of the PCC.

Women from tribal areas seek help from Bhagat (local spiritual healer) if they are not conceiving. Bhagat asks them to keep fast and worship the goddess. Also, prescribes herbal medicines. Women were not aware of the blood group and its significance. Women are also unaware of sexually transmitted diseases. Women are generally unaware of when to seek health care.

However, they are aware that overweight or obese women may have difficulty while conceiving. Women also explicitly said that if the husband does not communicate well with his wife, then planning a child does not make any sense as it is a mutual decision. “Husband should be sensible… if we say something, and what if he does not listen?” (2/2). Women are also aware that a low-weight mother gives birth to low-weight baby.

Women listed some of the do's and don'ts for a woman who wants to conceive:

“Not to eat tobacco” (1/5)

“Do not apply Mishri (roasted tobacco applied on teeth/gums)” (3/5)

“Do not fast” (5/5)

“Not to eat spicy food” (6/5)

**Figure 2:**
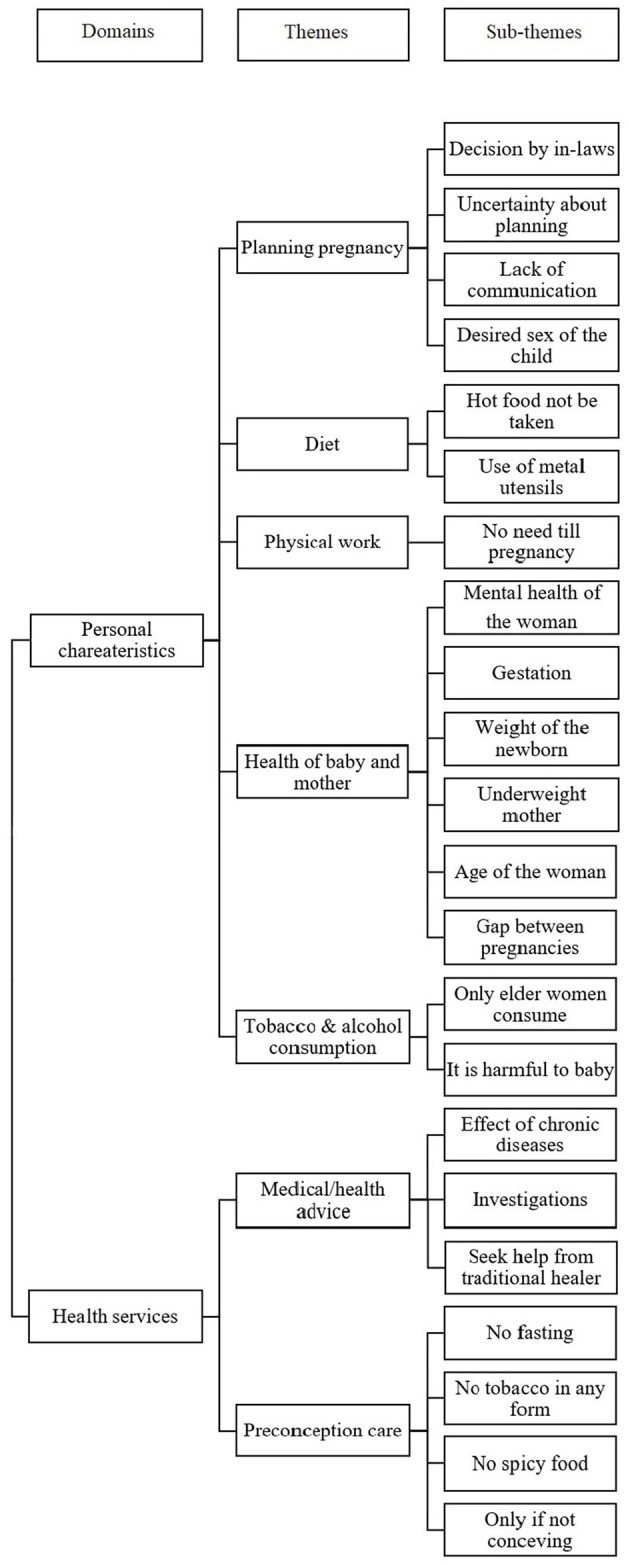
Emerged themes and sub-themes after focus group discussions.

Figure 2 depicts in a usual format the themes and subthemes that emerged after analysis (8).

There was no difference between the two groups regardless of the proposed intervention.

## Discussions

The study participants were only from rural areas, including two tribal blocks. Considering the one million population and 539 villages of these four blocks, we conducted two FGDs per block. The women in these groups were more or less having the same age and educational level, hence, disaggregation by age or education to control for its differing effects was not necessary. It was not intended also. The study showed that overall knowledge about PCC is low among women desiring pregnancy within 1 year. A few of them practiced behavior associated with optimizing the BMI, improving hemoglobin levels and very few accessed services related to PCC. The PCC is not rolled out in the government and the private health sector as yet. Health promotion, self-care may be necessary for promoting PCC (9). Many studies on PCC conducted among women visiting hospitals also reported similarly low levels of knowledge (10–16). In a study in the United States of America (USA), lack of knowledge was identified as the most crucial barrier to the practice of preconception behavior, the second most crucial barrier was the cost (17). China is a world leader in the implementation of PCC, covering its entire rural population. National Preconception Health Care Project was scaled up to all the rural areas of the country since 2013, and all couples planning pregnancy within 6 months were assessed for risk factors using an ABCDX category of preconception risk factors. It was found that 68.29% of couples had one or more preconception risk factors. The spectrum included avoidable risks like smoking and alcohol consumption; manageable through medical interventions like anemia, reproductive tract infections in women, abnormal liver or renal functions in men; controllable risk factors including diseases and conditions that cannot be cured, but risk factors can be modified (18). In India, PCC is largely restricted to contraceptives for family planning purposes and peri-conceptual supplementation of folic acid, rather than for promoting land of woman and man, and health of the newborn.

### Planning of Pregnancy

The mother-in-law influences the decision for planning the first pregnancy. However, the couples have greater decision-making power for planning subsequent pregnancies. In India, it is estimated that in 2015, out of 48.1 million pregnancies, 48% were unintended; of which, 33% were aborted, 5% were miscarriages from unintended pregnancies, and 11% were unintended births (19). Unintended pregnancies every year result in 25 million unsafe abortions and 47,000 maternal deaths globally, as per estimates of WHO (20). Implementation of comprehensive PCC will go a long way for preventing unintended pregnancies, thereby reducing the burden on women for abortion and thus prevent maternal ill-health and deaths due to unsafe abortion.

### Nutrition and Body Weight

Women were knowledgeable about the intake of various types of food groups. However, they did not know about the appropriate weight, when they should conceive. The women were not aware of the use of multiple micronutrients for preventing adverse pregnancy outcomes. In the USA, women had better knowledge about folic acid supplementation before pregnancy (15).

### Tobacco and Alcohol Consumption

In Kenya, most women know that they should avoid alcohol both before pregnancy and during pregnancy (10). In the USA, women know about alcohol and drug consumption risk during the preconception period (15). The discussions pointed out that the habit of tobacco consumption is decreasing among young girls, which is in concordance with national-level surveys (19).

### Medical Advice Before Pregnancy

Women have limited knowledge about the effect of chronic medical conditions like diabetes and hypertension on pregnancy planning and thereby on the health of the newborn (18). Contrarily in another study, many women opined that they must seek treatment for anemia, obesity, and diabetes before pregnancy, and hypertension before or during pregnancy causes miscarriage (10).

### Preconception Services: Knowledge and Utilization

Knowledge or awareness about PCC differs widely in different countries. A systematic review of 34 studies from 2000 to 2017 from 14 countries revealed that the tools assessing preconception health knowledge usually focus on fertility, folic acid supplementation, alcohol consumption, mental health, and some issues about the health of men (13, 21, 22). Another systematic review of studies done during 1998 and 2008 focusing on factors related to preconception health behaviors among childbearing age women in the United States, developed countries, and developing countries identified six major thematic areas: frequency of alcohol intake before and during pregnancy, glycemic control/diabetes management, physical activity before and during pregnancy, pregnancy planning behavior, cystic fibrosis carrier screening, and other risk factors (13, 21, 22). In the United Kingdom, women from different geographical regions have only modest knowledge (23). Women in the USA have low to moderate knowledge of issues related to preconception health (24). The proportion of women knowing PCC varied from 7 to 85% in different countries (11, 12, 14, 15, 25–27). Usually, most of the women have a positive attitude toward seeking PCC (14, 16, 22, 26). About one-third of men and 60% of women in Jordan strongly agreed that parental health might seriously affect the health of the expected baby (28). In a study in South Ethiopia, about 20% of the women who just delivered were found to have good knowledge about PCC. In a study in Colorado, USA, young women have greater motivation for pregnancy prevention and contraception than promoting preconception health. In another study, participants agreed on improving preconception health, mostly among the predominantly low-income group, the Mexican American population in the United States. Most of the desired information is from obstetricians (15). There was a lack of knowledge about PCC among women having chronic non-communicable diseases and a lack of intent for preconception health promotion and pregnancy planning among women in Hershey (18). About 15% of women were aware of preconception folic acid supplementation in North-western Ethiopia (29). The highest knowledge was about Reproductive and Child Health (RCH) risk factors and the lowest about health promotion (27). Most women have average knowledge of PCC, including verbal, physical, and sexual abuse (15).

Irrespective of knowledge and attitudes, only about 32 to 50% of women seek PCC in Nepal, Nigeria, Malaysia, and Iraq (11, 13, 14, 26).

Barriers include lack of social support and lack of awareness; women opined that the objectives of the program might be promoting healthy babies rather than preventing unhealthy babies (30).

Knowledge on PCC is usually found to be associated with education, age of the woman, number of children, family planning measures, receipt of information, chronic health problems, and monthly income in Zambia, Northwest Ethiopia, and Nepal (12, 25, 29, 31).

PCC is comparatively new in India. The women consulting obstetricians may receive some information and services. Federation of Obstetric and Gynecological Societies of India in 2016 formulated the National PCC guidelines for their members recommending actions are based on the strength of recommendation and extent of evidence for providing PCC to women. However, only a few private practitioners comprehensively provide PCC in India (32). In the study area, there are no specialists.

Readers should view all these findings in the background of rural and tribal women who are poor, have low educational levels, and lack access to the services of specialists. The government of India is already planning the uniform implementation of PCC. In the background of findings of this study, the government must pay attention to three major aspects: the contents, communication with in-laws including husband, and counseling training to all frontline health workers, including ASHAs. These activities should go parallel to the provision of medicines and equipment. The financial implications are not high compared to the potential accrued benefits.

### Strengths and Limitations

The moderators and facilitators are experienced and qualified personnel. They conducted the FGDs in the local language (Marathi) and are fluent in that language. There were no distractors. All eligible women from selected villages were invited. The findings are representative of women from an entire district population of more than 6 million.

The mothers-in-law were not included in the study. The group of participants mainly consisted of women having low levels of education. Emphasis on preconception health of men was almost lacking. The place of FGD was a subcenter building, which may affect their expressions. We conducted the study in rural and tribal settings; the findings may not represent the urban areas.

## Conclusions

The women had some knowledge about antenatal care only. About 50% of the pregnancies were planned. The women had very little knowledge about PCC, and service utilization was unsatisfactory. No participant knew about specific health conditions needing treatment before conception and their adverse impact on the health of the newborn. The participants were aware of some hazards of tobacco and alcohol consumption. A small proportion of participants had an addiction to tobacco and alcohol, but men in the family had. The behavior of smoking and alcohol consumption of the husband was not considered as a risk for pregnancy. There is an urgent need to educate the community about PCC and provide PCC services to promote maternal and newborn health and prevent maternal and newborn mortality.

## Data Availability Statement

The raw data supporting the conclusions of this article will be made available by the authors, without undue reservation.

## Ethics Statement

Institutional Ethics Committee (DCGI Regd. No. ECR/313/Inst/MH/2013/RR-16) approved the study vide reference number; BVDUMC/IEC/11 Dated; 30-04-2018. The patients/participants provided their written informed consent to participate in this study.

## Author Contributions

PD, JG, PP, SP, AP, MK, AS, and KB designed the concept of assessment of the PCC programme in Nashik District. PD, JG, PP, SP, and AC conducted the FGDs. AD and MK helped to assemble women. PD, JG, and AC performed data analysis. PD wrote the first draft of the manuscript. JG and KB revised the manuscript extensively. All authors approved the final version for publication. All authors have the appropriate permissions and rights to the reported data.

## Funding

This study was supported by UNICEF Maharashtra through the Directorate of Health Services, Government of Maharashtra.

## Conflict of Interest

The authors declare that the research was conducted in the absence of any commercial or financial relationships that could be construed as a potential conflict of interest.

## Publisher's Note

All claims expressed in this article are solely those of the authors and do not necessarily represent those of their affiliated organizations, or those of the publisher, the editors and the reviewers. Any product that may be evaluated in this article, or claim that may be made by its manufacturer, is not guaranteed or endorsed by the publisher.
